# Gadolinium retention within multiple rat organs after intra-articular administration of gadolinium-based contrast agents

**DOI:** 10.1007/s00256-020-03695-3

**Published:** 2021-01-06

**Authors:** Michael D. Ringler, Nicholas G. Rhodes, Jennifer R. Ayers-Ringler, Daniel R. Jakaitis, Robert J. McDonald, David F. Kallmes, Jennifer S. McDonald

**Affiliations:** 1 Department of Radiology, Mayo Clinic, 200 First Street SW, Rochester, MN 55905 USA; 2 Department of Neurosurgery, Mayo Clinic, 200 First Street SW, Rochester, MN 55905 USA

**Keywords:** Gadolinium, Arthrography, Magnetic resonance imaging, Contrast media, Brain

## Abstract

**Objective:**

To characterize the extent of retention and biodistribution of gadolinium (Gd) following intra-articular (IA) injection of linear and macrocyclic gadolinium-based contrast agents (GBCAs) into the knee joint of a rat model.

**Materials and methods:**

Fifteen Wistar rats were divided into five groups and underwent fluoroscopically-guided injections of both knee joints of (1) clinical 1:200 dilution (low dose, LD) gadodiamide (linear GBCA), (2) LD gadobutrol (macrocyclic GBCA), (3) undiluted (high dose, HD) gadodiamide, (4) HD gadobutrol, and (5) saline. Gd concentrations were quantified by inductively coupled plasma mass spectrometry in (1) blood and urine samples obtained over a 72 h period and (2) knee joint tissues, brain, kidney, and bone marrow at 3 days post-injection.

**Results:**

Both HD and LD gadodiamide and gadobutrol were rapidly absorbed from the joint with peak serum and urine concentration at 1 h post-injection, with relatively faster clearance of gadobutrol. All GBCA-exposed groups had detectable levels of Gd in the joint tissues, bone marrow, and/or kidneys (median tissue gadolinium range: 0.1–71 μg Gd/g tissue), with higher amounts observed with gadodiamide versus gadobutrol. Retention within brain tissues was only detected following HD gadodiamide administration but not LD gadodiamide nor HD or LD gadobutrol.

**Conclusion:**

There was rapid systemic absorption, redistribution, and widespread multi-organ retention of Gd following IA injection of both linear and macrocyclic GBCAs, despite substantial amounts of urinary excretion. Higher concentrations of Gd were observed with administration of gadodiamide compared to gadobutrol in most tissues and biofluids.

## Introduction

Retention of gadolinium (Gd) within various animal tissues following intravenous (IV) administration of gadolinium-based contrast agents (GBCAs) has been documented in preclinical studies since 1984 [1]. However, it was not until 2006, when nephrogenic systemic fibrosis was first reported that tissue retention of Gd became of elevated clinical concern [2]. Most GBCAs widely distribute within the vascular and extracellular spaces after IV administration and are predominantly renally excreted [3]. Tissue retention has now been reported throughout the body, including the brain, kidney, liver, spleen, skin, and bone, even in patients with normal renal function [4–7]. As a result, regulatory organizations including the US Food and Drug Administration have implemented restrictions and warnings regarding IV GBCA use [8, 9].

Intra-articular (IA) injection of diluted GBCA is a commonly employed practice to improve visualization of the cartilage, joint space, and soft tissues during MR arthrography. This common off-label use of GBCA is considered safe [10]. However, few studies have evaluated the interactions of Gd in the body following IA injection. An early study by Engel et al. [11] demonstrated retention of Gd into the synovial membrane after IA injection of gadopentetate dimeglumine (Magnevist) into human knees, and transient retention into the articular cartilage in rabbits. Swift elimination of Gd from the joint, generally over several hours, has been shown in a study in rabbits [12]. Similar rapid elimination is suggested in humans, with studies demonstrating no MR signal alteration from IA GBCA 6 h after injection, presumably due to systemic absorption via the joint synovium [10, 13, 14].

Two recent clinical studies [15, 16] attempted to ascertain if MR arthrography resulted in intracranial Gd retention. Both looked for T1-weighted signal abnormalities on MR brain exams, similar to prior IV GBCA studies, following one to three IA injections of GBCAs. No signal abnormalities were observed; however, MR imaging analysis is known to have limited sensitivity at detecting retained Gd. Inductively coupled plasma mass spectrometry (ICP-MS) is considered the more sensitive reference standard for quantifying Gd in biofluids and tissues [17]. Our study seeks to characterize the extent of retention and biodistribution of Gd following IA injection of linear and macrocyclic GBCAs into the knee joint of a rat model [5]. We hypothesize that Gd should redistribute broadly throughout the various organs and tissues of the body after IA GBCA administration.

## Materials and methods

All animal protocols were reviewed, approved, and overseen by the Mayo Clinic Institutional Animal Care and Use Committee.

### Animals

Fifteen healthy adult (420–450 g) male Wistar rats (Charles River Laboratories, Wilmington, MA) were housed under standard laboratory conditions, with food and water provided ad libitum. Rats were divided into groups of three, with all co-housed animals receiving the same injectate.

### Joint injection

The rats received monitored anesthesia by 1–2% vaporized isoflurane. Both stifle joints of each mouse were shaved and then prepped in a sterile manner. Under fluoroscopic and tactile guidance by an MSK radiologist (NGR, 8-year experience with humans), a 27-gauge TB needle was passed into the stifle joint. After placement, the needle hub was filled with the injectate (described below) and a slip tip, 1-cc tuberculin syringe (both needle and syringe from Cardinal Health, Dublin, OH, USA) containing additional injectate was attached to the needle. The injection was performed under fluoroscopic visualization, with distention of the joint capsule visualized. During no injection was extra-articular or intravascular injectate visualized. Two hundred-microliter total volume of injectate was placed into each knee. This volume endpoint was chosen during injection of the first mouse as one that would distend the joint without causing rupture or causing high back pressure on the syringe. This held true for all injections. Direct pressure was held at the needle site for 1 min beginning immediately upon removal of the needle.

All rats had both knees injected at a single time point. Rats were treated with IA injections of 0.5 mmol/ml gadodiamide (Omniscan; GE Healthcare), 1 mmol/ml gadobutrol (Gadavist; Bayer), or normal saline. Two GBCA doses were utilized for this study: a low dose (LD) to approximate the amount of GBCA used in adult humans for MR arthrography, and a high dose (HD) to maximize total GBCA administered. The LD group received the standard clinical 1:200 dilution of GBCA used for MR arthrography, diluted into a 1:1 solution of iohexol (Omnipaque 300; GE Healthcare) iodinated contrast material and saline. Iodinated contrast was used to visualize the injection under fluoroscopic guidance and verify no extracapsular leakage. The HD group received only undiluted GBCA, as undiluted GBCA attenuates x-rays similar to iodinated contrast material. The resultant injected concentrations of GBCA were 2.5 μmol/ml in the LD gadodiamide group, 5 μmol/ml in the LD gadobutrol group, 0.5 mmol/ml in the HD gadodiamide group, and 1 mmol/ml in the HD gadobutrol group. Total injected gadolinium into each mouse, after injection of both knees, in each group was 1 μmol, 2 μmol, 0.2 mmol, and 0.4 mmol, respectively. The saline injectate consisted of a 1:1 dilution of iodinated contrast and saline.

### Biofluid harvesting

Blood serum was collected by jugular venipuncture using a 23–25 gauge beveled needle and 1-ml syringe by DRJ (10-year experience). Briefly, the rat was anesthetized using vaporized isoflurane at 3% mixed with room air and maintained with vaporized isoflurane at 1–2%. Blood was then directly ejected gently into a sterile 1.5-ml microcentrifuge tube and allowed to clot at room temperature for at least 30 min before separating out the serum by centrifugation at 1500×*g* for 5 min at room temperature. Serum was transferred using sterile pipet tips to new 1.5-ml microcentrifuge tube. Urine was collected by free catch as the rat recovered from the anesthesia given during blood draw, or from metabolic cages. Metabolic cages were thoroughly cleaned using the cage wash and were visually inspected carefully before each use for debris. Gloves were changed frequently to prevent cross-contamination of samples. Blood serum and urine samples were collected at baseline, 1 h, 3 h, 6 h, 24 h, and 72 h following injection.

### Tissue processing

Three days after IA injections, rats were anesthetized with a sub-lethal, weight-dependent injection of 100 mg/kg pentobarbital, then euthanized via exsanguination and perfused with 10% neutral buffered formalin. Following euthanasia, brain, femur, stifle (knee) joint, and kidney tissues were harvested via necropsy and fixed in Trump’s Fixative (4% paraformaldehyde +1% glutaraldehyde in 0.1 mol/l phosphate buffer) for at least 3 days with gentle agitation, then transferred to 10% neutral buffered formalin for long-term storage. For brain samples, the deep cerebellar nuclei were microdissected from one hemisphere in each rat. For kidney samples, a portion of the medulla was harvested from one kidney in each rat. For joint tissue samples, the complete stifle (knee) joint, including cartilage, synovium, surrounding muscle/tendon, connective tissues, inferior femoral bone, and superior tibial bone about the knee was harvested. For bone marrow samples, a portion of the superior femur, distant from the injected joint, was harvested. All necropsies and dissections were performed by JRA (10-year experience).

### Gadolinium analysis

Gd was quantified by the Mayo Clinic Metals Laboratory using ICP-MS as shown by an earlier investigation [18]. The lower limit of detection for this assay is 0.1 ng Gd/ml for biofluids and 0.1 μg Gd/g for tissue.

### Statistical analysis

All statistical analyses were performed by JRA (8-year experience) and JSM (13-year experience) using JMP (version 13, SAS Institute) and Prism (version 8.2.1, GraphPad). Median and range of continuous data are presented due to non-normal distribution. Differences in Gd concentration between groups were assessed using the Wilcoxon rank sum test. Significance was assigned to differences of *P* ≤ 0.05.

## Results

### Biofluid analysis

**Fig. 1:**
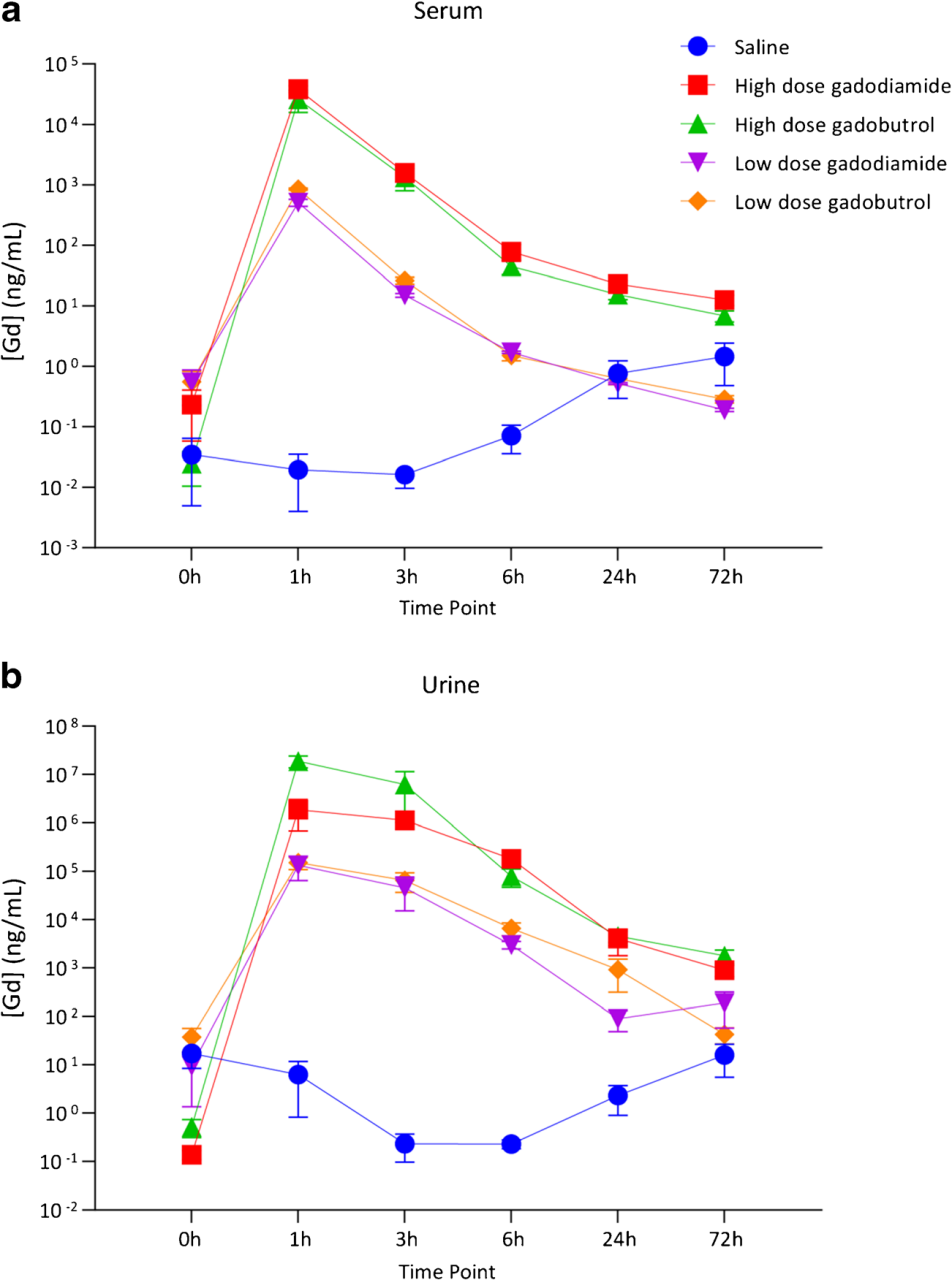
Elemental gadolinium (Gd) levels in the serum (**a**) and urine (**b**) as determined by inductively coupled plasma mass spectrometry. Data are presented as the concentration in ng Gd/ml fluid (median ± 95% CI)

Comparisons of the amounts of Gd detected by ICP-MS at various time points in blood serum and urine samples following IA injection of GBCA are shown in Tables 1 and 2. All GBCA-exposed groups demonstrated peak Gd concentration in the serum and urine at 1 h post-injection, followed by rapid washout beginning at about 3 h (Fig. 1). Serum Gd levels in the LD gadodiamide and LD gadobutrol groups were not significantly different from levels in the saline control group at 24 h post-injection (*P* = 0.51 and *P* = 0.83, respectively). In comparison, significantly higher serum levels of Gd compared to the saline group persisted to 72 h post-injection following HD gadodiamide (*P* = 0.0495) and HD gadobutrol (*P* = 0.0495) injections. Urine Gd levels in HD gadodiamide- and HD gadobutrol-exposed groups remained significantly higher than the saline group at 72 h post-injection (*P* = 0.0495 for both groups), while urine levels in LD gadodiamide- and LD-gadobutrol-exposed groups were non-significantly higher than the saline group (*P* = 0.13 and *P* = 0.28, respectively). At 1 h post-injection, serum Gd levels were higher in gadodiamide-exposed vs. gadobutrol-exposed rats (median serum values = 34,696 ng/ml for HD gadodiamide vs. 17,621 ng/ml for HD gadobutrol), and urine Gd levels were higher in gadobutrol-exposed vs. gadodiamide-exposed rats (median urine values = 1,854,114 ng/ml for HD gadodiamide vs. 23,820,000 for HD gadobutrol), ostensibly reflecting the relatively slower clearance of gadodiamide compared with gadobutrol.

**Table 1:** Serum gadolinium (Gd) concentrations at various time points following intra-articular injection

	ng Gd/ml serum, (median and range)					
Group	0 h	1 h	3 h	6 h	24 h	72 h
Saline	0.01 (0.00–0.09)	0.02 (0.00–0.04)	0.02 (0.00–0.02)	0.09 (0.00–0.12)	0.67 (0.00–1.62)	0.59 (0.38–3.41)
High-dose gadodiamide	0.09 (0.03–0.58)	34,696 (32742–48,306)	1158 (1075–2555)	78.76 (36.78–120)	25.26 (16.54–27.51)	14.43 (7.69–15.75)
High-dose gadobutrol	0.01 (0.01–0.05)	17,621 (14354–46,869)	1723 (278–2000)	44.92 (24.26–65.55)	16.5 (10.32–19.36)	6.67 (4.53–9.38)
Low-dose gadodiamide	0.49 (0.02–1.13)	566 (374–595)	15.59 (13.03–16.65)	1.66 (1.62–1.86)	0.51 (0.49–0.56)	0.19 (0.17–0.21)
Low dose gadobutrol	0.55 (0.09–1.03)	883 (778–898)	25.35 (21.08–32.71)	1.34 (1.15–2.03)	0.61 (0.41–0.87)	0.31 (0.21–0.34)

**Table 2:** Urine gadolinium (Gd) concentrations at various time points following intra-articular injection

	ng Gd/ml urine, (median and range)					
Group	0 h	1 h	3 h	6 h	24 h	72 h
Saline	21.62 (0.21–29.64)	1.30 (0.35–17.07)	0.23 (0.10–0.37)	0.24 (0.15–0.30)	1.93 (0.08–4.92)	6.16 (4.89–36.96)
High-dose gadodiamide*	0.12 (0.10–0.20)	1,854,114 (689986–3,018,242)	1,124,178 (0)	181,373 (0)	2578 (1099–8489)	1087 (424–1168)
High-dose gadobutrol	0.55 (0.15–0.86)	23,820,000 (8670740–24,000,000)	1,229,390 (803300–16,653,400)	88,892 (19029–129,453)	4344 (2553–6787)	2167 (696–2491)
Low-dose gadodiamide	2.25 (0.48–25.68)	67,346 (61441–272,132)	45,552 (15071–76,032)	2711 (2233–3993)	98.54 (14.26–158)	95.14 (23.45–448)
Low-dose gadobutrol	45.56 (1.57–65.69)	141,136 (82913–227,951)	83,833 (9477–102,339)	6913 (3063–9801)	627 (48.69–2075)	28.68 (25.26–73.75)

*Urine was unable to be collected from two of the three rats in this group at the 3 h and 6 h time points

### Tissue retention of gadolinium

Relative to control rats, ICP-MS analysis revealed both HD gadodiamide and HD gadobutrol-exposed rats had elevated levels of elemental Gd in the joint tissues, bone marrow, and kidneys at 3 days post-injection (Median Gd levels: Joint tissue: HD gadodiamide = 2.0 μg Gd/g tissue, HD gadobutrol = 0.5 μg/g, LD gadodiamide = 0.2 μg/g; Bone marrow: HD gadodiamide = 1.5 μg/g, HD gadobutrol = 0.1 μg/g, LD gadodiamide = 0.1 μg/g; Kidney: HD gadodiamide = 71 μg/g, HD gadobutrol = 14 μg/g, LD gadodiamide = 1.2 μg/g, LD gadobutrol = 0.2 μg/g) (Table 3). No Gd was detected in the joint tissue or bone marrow of LD gadobutrol rats. In descending order, HD gadodiamide had the highest median amount of Gd retention in all tissues, followed by HD gadobutrol, LD gadodiamide, and LD gadobutrol (Table 3). Detectable concentrations of Gd were highest in the kidneys, followed by joint tissue, and then bone marrow for both types and doses of GBCA tested. Only HD gadodiamide-exposed rats demonstrated detectable levels of elemental Gd in the brain (median Gd level 0.2 μg/g), (Table 3). Gd levels in neural tissues from other GBCA-exposed groups were below the assay limit of detection.

**Table 3:** Tissue gadolinium (Gd) concentrations 3 days following intra-articular injection

	μg Gd/g tissue, (median and range)			
Group	Joint	Kidney	Bone marrow	Brain
Saline	**<** 0.1 (LOD)	**<** 0.1	**<** 0.1	**<** 0.1
High-dose gadodiamide	2.0 (0.7–4.6)	71 (65–89)	1.5 (0.2–2.1)	0.2 (0.1–0.2)
High-dose gadobutrol	0.5 (0.5–0.6)	14 (11–20)	0.1 (0.1–0.1)	**<** 0.1
Low-dose gadodiamide	0.2 (0.2–0.3)	1.2 (1.0–1.4)	0.1 (0.1–0.1)	**<** 0.1
Low-dose gadobutrol	**<** 0.1	0.2 (0.1–0.3)	**<** 0.1	**<** 0.1

*LOD*, assay limit of detection 0.1 μg Gd/g tissue

## Discussion

This animal model study demonstrated that there is rapid systemic absorption, redistribution, and widespread multi-organ retention of Gd following IA injection of GBCAs, despite evidence of rapid first-pass renal elimination. Within the limits of temporal resolution, IA administration of both gadodiamide and gadobutrol resulted in peak blood serum Gd levels in 1 h with both undiluted and clinically relevant diluted GBCA doses. Consistent with studies regarding retention of Gd following IV administration, this investigation also reveals GBCA class and dose dependency [5, 19]. After 3 days, Gd retention was observed in the tissues immediately around the injected joint, in the bone marrow, and in the kidneys, even at the 1:200 clinical dilution used for MR arthrography. The higher undiluted dose demonstrated higher amounts of tissue retention, including within the deep cerebellar nuclei. This suggests that Gd biodistribution and retention following the routine off-label use of GBCA for MR arthrography are systemically similar to IV injection of GBCA.

In their initial study, Kanda et al. [4] state that they noted increased high-signal in the dentate nucleus and globus pallidus in patients with history of multiple IV GBCA administration. Indeed, their initial study reviewed only patients with a history of six prior GBCA-employed examinations. A subsequent paper by Kanda et al. [20] suggests that three to four IV doses of linear GBCAs are necessary to elevate T1-signal above baseline noise. The standard IV dose is generally between 5 and 10 ml in a 60–80-kg adult human. In a routine MR arthrogram, contrast dose in a knee is approximately 20 to 40 ml of a 1:200 dilution of the same GBCA, around 100-times less than that of IV administration. It is therefore not surprising that two recent studies were unable to detect retained Gd in the brain using T1-weighted signal on MRI in patients who had received only one to three MR arthrograms [15, 16]. In our study, intracranial Gd retention was detected using ICP-MS only following IA injection of undiluted gadodiamide. However, as even the most sensitive state of the art analytic instrumentation have well-established lower limits of detection, it is likely that extremely small amounts of Gd that are currently undetectable are retained in a dose-dependent manner, even with diluted GBCA administration for both linear and macrocyclic agents.

Residual Gd was observed in all knee joints, except following 1:200 diluted gadobutrol injections. Rahmouni et al. [21] studied periarticular bone in dogs after IA injection with Gd-DOTA (a macrocyclic agent) and found Gd retention in bone at 2 h post-injection, but not later. This would fit with the observed trends in our study concerning macrocyclic agents. In fact among rats treated with the 1:200 diluted gadobutrol, with the exception of the kidneys, none of the other tested organs demonstrated detectable Gd retention. While there is currently no evidence that Gd retention resulting from IA GBCA administration causes any harm, this GBCA class difference may require further study in light of the fact that regulatory agencies such as the US Food and Drug Administration still allow IA administration of linear GBCAs [8, 9]. Our rat model suggests that despite the much lower dose of administered GBCA in MR arthrography, there is still potential tissue retention when using a linear GBCA.

Our study has several limitations. First, we examined a small number of rats. It is likely that larger numbers would show more significance and more variation in the results. However, the goal of this initial investigation was to simply confirm Gd retention following IA GBCA injection. Second, we were limited to a single time point of tissue sampling for analysis, preventing us from examining the potential distribution curve of Gd throughout the body and brain over time and extent of chronic Gd retention following IA administration. Third, we injected both knee joints in the same time period and did not perform a single knee injection that would serve as the equivalent of a single MR arthrogram. The rat knee joint is large enough for consistent access by needle and allows for maximization of administered IV volume. While this decision was made to maximize the chances of detecting Gd, two MR arthrograms on the same patient are still clearly within the realm of clinical practice. Fourth, as we only examined two GBCAs, we cannot globally extend our findings to all agents. Finally, translating preclinical animal model results to human patients is always uncertain and the amounts of retained Gd following IA administration in humans may differ due to more complex biodistribution and/or clearance. Additional studies in both animals and humans to further examine the pharmacokinetics and biodistribution of retained Gd following IA GBCA injection would be helpful.

In conclusion, our rat model demonstrates that Gd is rapidly absorbed into the blood stream in a dose-dependent manner following IA injection of linear and macrocyclic GBCAs. IA Gd is predominantly excreted in the urine, but a small amount is retained in joint, bone marrow, kidney, and brain tissues. Although higher concentrations of retained Gd were observed following administration of low-dose gadodiamide when compared to gadobutrol, tissue retention in the kidneys was observed even with IA administration of diluted gadobutrol. Such findings challenge our understanding of the biodistribution of pharmacologic agents administered into IA spaces and suggest that in the case of GBCA use, IA and IV administration appears to be equivalent in their capacity to lead to retention of Gd into mammalian tissues.

## Abbreviations and Acronyms

Gd: Gadolinium
IA: Intra-articular
GBCA: Gadolinium-based contrast agent
HD: High dose (undiluted)
LD: Low dose (clinical 1:200 dilution)
IV: Intravenous
ICP-MS: Inductively coupled plasma mass spectrometry
